# Community-Defined Challenges: A Five-Year Qualitative Needs and Resources Assessment in Vulnerable Latino Populations of Miami-Dade County

**DOI:** 10.3390/ijerph23050546

**Published:** 2026-04-23

**Authors:** Gira J. Ravelo, Michelle Robinson, Gladys Ibañez, Mariana Sanchez, Arnaldo Gonzalez, Beatriz Macias Gomez-Estern, Patria Rojas, Mario De La Rosa, Victoria Behar-Zusman

**Affiliations:** 1 CRUSADA, Robert Stempel College of Public Health and Social Work, Florida International University, Miami, FL 33199, USA; gonzalea@fiu.edu (A.G.); delarosa@fiu.edu (M.D.L.R.); 2 Health Promotion & Disease Prevention, Robert Stempel College of Public Health and Social Work, Florida International University, Miami, FL 33199, USA; mfrobins@fiu.edu (M.R.); msanche@fiu.edu (M.S.); proja003@fiu.edu (P.R.); 3 Epidemiology, Robert Stempel College of Public Health and Social Work, Florida International University, Miami, FL 33199, USA; gibanez@fiu.edu; 4 Department of Social Anthropology, Basic Psychology and Public Health, Pablo de Olavide University, 41013 Seville, Spain; bmacgom@upo.es; 5 School of Nursing & Health Studies, University of Miami, Miami, FL 33146, USA; vbehar-zusman@miami.edu

**Keywords:** SAVA syndemic, community-based participatory research (CBPR), Latino health disparities, qualitative needs assessment

## Abstract

**Highlights:**

**Public health relevance—How does this work relate to a public health issue?**

- This study addresses the “SAVA” syndemic—the synergistic interaction of substance use, violence, and HIV/AIDS—which disproportionately impacts marginalized Latino populations in Miami-Dade County, a region with the highest HIV rates in Florida.
- It highlights how localized “micro-communities,” such as farm-workers, inner-city residents, and LGBTQ+ individuals, experience distinct health crises that are often masked by aggregated county-level epidemiological data.

**Public health significance—Why is this work of significance to public health?**

- The research uncovers hidden public health burdens, including methamphetamine-linked sex trafficking in LGBTQ+ networks, rampant youth vaping in inner cities, and an opioid crisis within farm-working communities.
- It identifies critical gaps in mental health service utilization, revealing that mental health issues are frequently masked by substance use and suppressed by cultural stigmas and institutional fears.

**Public health implications—What are the key implications or messages for practitioners, policy makers and/or researchers in public health?**

- Practitioners and policy makers must consider more than “zip-code level” research and supplement such findings with localized, community-defined assessments to ensure resources and interventions are culturally rooted and reach the most vulnerable sub-populations.
- Effective public health strategies require a collaborative, multi-sectoral model that bridges organizational silos to address the interconnected nature of substance use, violence, HIV risk, and mental health.

**Abstract:**

Background: Miami-Dade County ranks first in Florida for HIV cases, yet broad epidemiological data often masks the “on-the-ground” reality of its most vulnerable residents. While standard reports suggest declining domestic violence, these statistics fail to account for community-defined health crises—the “SAVA” syndemic (substance use, violence, and HIV/AIDS)—occurring within localized micro-communities. Methods: Leveraging five years of Community-Based Participatory Research (CBPR) and Grounded Theory, this study engaged 97 community members and leaders to unmask these hidden burdens. We employed a multi-level sequential design and methodological triangulation, incorporating community forums, focus groups, and interviews with farm-workers, inner-city residents, and LGBTQ+ individuals. Results: Findings reveal a disconnect between official data and community reporting, including “Party and Play” methamphetamine/sex-trafficking networks in the LGBTQ+ scene, rampant youth vaping in inner cities, and child sexual abuse and opioids in farm-working communities. Mental health emerged as a pervasive need, masked by substance use and suppressed by cultural stigmas and institutional fears. Conclusions: Findings from this study highlight the value of community-level approaches in generating localized, culturally grounded insights that may not be fully captured in more aggregated geographic analyses (e.g., zip code, county, or state levels). We propose a collaborative, multi-sectoral model to address the systemic factors underlying the SAVA syndemic in these communities.

## 1. Introduction

### 1.1. Latinos in South Florida

Latinos constitute the largest ethnic minority group in the United States, with over 65 million individuals, representing 19.5% of the U.S. population [1], and accounting for 33% of the immigrant population [2]. While the distribution of Latinos is concentrated predominantly within California, Texas, and Florida [3], Miami-Dade County (MDC), Florida, stands out. According to 2022 U.S. Census data, over 69% of MDC’s residents identify as Latino or of Latino descent [4]. The rich diversity within MDC encompasses various Caribbean, South, and Central American origin Latino groups, with over 75% of residents speaking a language other than English, primarily Spanish (66.6%) [5]. These demographic characteristics are critical when developing intervention and prevention efforts targeting MDC’s Latino community.

Health disparities impacting Latino communities are compounded by elevated rates of poverty, substance use, and limited access to healthcare services [5,6]. Furthermore, MDC faces significant public health concerns, ranking first in the state for HIV cases [7]. Violence, including intimate partner violence (IPV) and mental health issues, which spiked between the years 2008 to 2018 [8,9], remain pressing concerns, particularly among Latino communities of MDC.

Despite existing research examining substance use, violence, HIV risk, and mental health among Latino populations, research has largely focused on these issues in isolation or at aggregated geographic levels (e.g., county or state). As a result, there remains a limited understanding of how these conditions intersect within a syndemic framework at the level of localized, marginalized “micro-communities,” particularly among diverse Latino subpopulations such as farm-working, low-income inner-city, and LGBTQ+ communities within a single geographic region. This gap is especially critical in Miami-Dade County, where broad epidemiological data may obscure important within-group differences and community-specific needs. The present study addresses this gap by employing a multi-level community-based participatory research (CBPR) approach to examine substance use, violence, HIV risk, and mental health as interconnected phenomena across distinct Latino communities.

The purpose of this study was to conduct a five-year, multi-community qualitative assessment to identify community-defined needs, resources, and service gaps related to substance use, violence, HIV risk, and mental health among marginalized Latino populations in Miami-Dade County. This study addresses a critical gap in the literature by providing localized, community-level insights into the intersection of these conditions within a syndemic framework, which are often not captured in broader epidemiological analyses. MDC offers a uniquely appropriate setting for this study due to its diverse and concentrated Latino population, which includes distinct subgroups such as farm-working, low-income inner-city, and LGBTQ+ communities. This diversity within a single geographic region allows for a comparative, multi-community perspective, allowing for the identification of both shared and community-specific needs, risks, and resources. Using a CBPR framework, this study also sought to enhance community capacity and inform multilevel, culturally grounded interventions aimed at improving health and well-being among diverse Latino populations in Miami-Dade County (MDC), particularly in relation to substance use, violence, and HIV/AIDS.

### 1.2. Latino Alcohol and Substance Use

Alcohol and marijuana are the two most common substances used. Both alcohol and marijuana use have been linked to acute and chronic health conditions. Alcohol is particularly prevalent among those with liver problems, hypertension, neurological and cardiac symptoms as well as cancer [10,11]. The Latino population is disproportionately affected by alcohol-associated health problems as well as alcohol-related arrests and accidents [12]. In the U.S., Latinos are more likely to abstain from alcohol use than Whites. Reports indicate that only 15.5% of whites abstain from lifetime alcohol use, while 31.8% of Latinos abstain from lifetime alcohol use [12]. However, Latinos who engage in alcohol use are more likely to engage in heavy or binge drinking, defined as 4 or more/5 or more (women/men) drinks on the same occasion [13].

Marijuana use has been linked to impairments in perception and cognition. Despite this, it is the most widely used non-opioid substance among young adults aged 18 to 25, followed by adolescents aged 12 to 17 [14]. Empirical research specifically on Latino marijuana use remains limited; however, some studies suggest that fear of deportation discourages marijuana use among undocumented Latinos [15]. Moreover, additional studies suggest that retention of certain heritage cultural norms and use of the Spanish language can serve as protective factors against substance use-related problems [13,16,17]. In fact, the 2024 Florida Youth Substance Abuse Survey (FYSA) [18] reported that although early substance use remains a concern among Florida’s youth, longitudinal data indicates a significant downward trend in early initiation. Between 2012 and 2024, high school students reporting alcohol experimentation at age 13 or younger decreased from 25.4% to 13.2%. Similarly, early marijuana use dropped from 11.7% to 5.1% during the same period [19].

However, indicators of acculturation, such as language acquisition and lower levels of origin cultural identity, are associated with an increased risk for substance use among Latinos [17,20,21], suggesting positive associations between acculturation and substance use.

### 1.3. Latinos and Violence

In the U.S., over 47% of women reported some form of contact sexual violence, physical violence, and/or stalking by an intimate partner at some point in their lives [22]. More comprehensively, intimate partner violence or IPV includes physical, emotional, and sexual harm, as well as stalking by either a former or current partner [23]. IPV has been associated with health, mental health, and substance use outcomes. For example, IPV has been associated with diabetes and respiratory and cardiovascular problems, as well as acute and chronic physical bodily pain, depression, and anxiety [24,25]. While IPV occurs among both men and women, women are more likely to experience a form of IPV (44.2% vs. 47.3%, respectively) [22]. While in MDC, overall reports slightly decreased, women represented 89.6% of reported sexual violence victims in 2024 [26]. Some empirical research suggests that Latinas are more likely to experience the negative impacts associated with IPV, such as poorer levels of mental health [27,28] and physical health [29,30].

In 2023, Vargas and colleagues [31] conducted a secondary data analysis of data from the Centers for Disease Control and Prevention’s Wide-ranging Online Data for Epidemiologic Research (CDC WONDER). Their findings indicated that between 2018 and 2021, Latino men experienced significantly higher firearm homicide rates compared to non-Hispanic White men across most age and gender categories. Specifically, the age-adjusted firearm homicide rate for Hispanic men was 2.43 times higher than that of non-Latino White males. While disparities were smaller for Latina women, their rates were still 1.17 times higher than their non-Latina White female counterparts. Moreover, the study found that these disparities were particularly pronounced in metropolitan counties, especially large central metropolitan areas, which also exhibited higher overall rates [31]. While there is emerging empirical research on violence within Latino communities, gaps remain in the literature to better understand the prevalence of violence among Latinos and the associated risk and protective factors that can serve to improve the health and wellbeing of this population.

### 1.4. Latinos and HIV/AIDS Risk

The United States has presented well-established evidence of racial and ethnic disparities concerning HIV diagnoses and prevalence [32]. Moreover, in MDC, the rate of new HIV diagnoses is significantly higher among Latinos (38.5 per 100,000) than among non-Hispanic Whites (26 per 100,000). Hispanic and Non-Hispanic Black residents together account for 88% of all diagnosed cases in the county [33]. These disparities extend beyond individual-level risk behaviors and are demonstrably influenced by social determinants of health (SDH) [34,35,36,37]. Consequently, alongside Black Americans, Latinos face an increased risk of HIV due to SDH such as economic hardship, limited healthcare access, lower educational attainment, racism, and stigma [32,36,38], as well as factors related to the physical environment [37,39]. For instance, Gant et al. [40] found that areas characterized by high levels of poverty, limited household incomes, and inadequate health insurance coverage exhibited the highest rates of HIV diagnoses among Latino populations. A comprehensive understanding of these community-level and social disparities is crucial for informing the expansion of HIV prevention and treatment services, thereby contributing to the overarching goal of minimizing new infections [41].

### 1.5. Latinos and Mental Health

Mental health remains a pertinent public health concern, with approximately 25% of the 18 and over population experiencing a mental health problem. Often, mental health and substance use problems co-occur; nearly 14% of the U.S. population aged 18 and older experienced both a mental health and a substance use disorder [14]. The mental health of the Latino populations in the U.S. is particularly concerning. Borges et al. [42] reported similar, though slightly higher, rates of suicide attempts among Hispanics (5.11%) compared to non-Hispanic Whites (4.69%) and non-Hispanic Blacks (4.15%). Despite the pronounced need for mental health services, Latinos are less likely to seek and receive mental health treatment, with less than half (35%) of those experiencing mental health challenges seeking care. In Miami-Dade, suicide ideation among LGBTQ+ Latino youth is particularly high, with one in four reporting attempts [6]. Research has speculated and generated support for potential barriers to Latino’s access to mental health services, including cultural factors and stigma, language barriers, legal status, and financial barriers related to poverty and insurance coverage [43].

## 2. Theoretical Frameworks

### 2.1. Community-Based Participatory Research (CBPR)

Community-Based Participatory Research (CBPR) is fundamentally a collaborative approach, emphasizing the integration of knowledge between community members and research investigators to foster a deeper understanding and effective response to phenomena impacting a community [44,45]. Key principles of CBPR include leveraging community strengths and existing resources and promoting shared learning through active collaboration among community entities and research investigators. This framework is particularly recommended when engaging with marginalized and minority populations [44,45]. For instance, a systematic review by Nueces et al. [46] found that CBPR trials achieved high success rates in recruiting and retaining minority participants and in demonstrating significant intervention effects. Beyond its effectiveness in fostering successful partnerships between researchers and community members in underserved communities, as described by Burklow and Mills [47] and Silka et al. [48], CBPR is also recognized as a promising approach for addressing mental health challenges in minority populations [49].

### 2.2. Grounded Theory (GT)

Grounded Theory (GT) comprises a systematic set of methods and processes that enable researchers to identify concepts and construct theories directly from qualitative data [50,51]. This approach is primarily inductive, guiding researchers from specific observations to general explanations of phenomena within the theory-generating process. There is broad consensus among researchers that knowledge derived from semi-structured and unstructured data has become “imperative for organizational strategic decision making.” In GT, qualitative interviews, such as those conducted in the current study, can be either semi-structured (where participants are asked a core set of open-ended questions designed to guide the interview) or unstructured (where questions are not predetermined prior to interviewing) [52].

## 3. Study Design

Researchers from Florida International University (FIU), in collaboration with the University of Miami (UM), received a Specialized Center Cooperative Agreement (U54MD002266) grant from the National Institute of Health (NIH), National Institute on Minority Health and Health Disparities (NIMHD). This funding established a multi-institutional, multidisciplinary research center, named The Center for Latino Health Research Opportunities (CLaRO), spanning both institutions within Miami-Dade County (MDC), Florida. CLaRO’s mission was to address the substance use, violence/trauma, and HIV/AIDS (SAVA) syndemic [53] impacting Latino populations in MDC.

CLaRO’s Community Engagement and Dissemination Core (CEDc) established a Community/Scientific Advisory Board (CSAB), comprised of community agency leaders and academic scientists from both UM and FIU. The establishment of and collaborations with the CSAB strictly adhered to Community-Based Participatory Research (CBPR) approaches. This ensured that research contributions from community leaders were integrated, relationships with community stakeholders and members were maintained, and optimal recruitment and engagement strategies were promoted. The collaborative efforts of the CEDc and CSAB included the planning and coordination of all community engagement and research-related activities.

**Figure 1:**
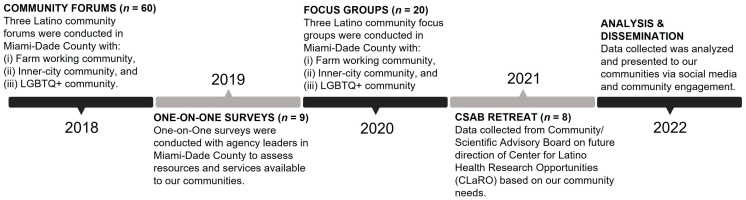
Participants and Community Assessment Timeline (N = 97).

The current study utilized sequential qualitative design applying strategies informed by the GT within a CBPR framework. The primary aim of the CEDc was to conduct a five-year multi-community-level assessment of SAVA-related needs, resources, and existing services among marginalized Latino communities in Miami-Dade County (MDC), FL (referred to as the “community assessment”) (Figure 1).

Essential to the successful completion of CLaRO’s community assessment was the combined 20 years of community engagement experience and vast networks and relationships with community partners held by UM and FIU. The CEDc and CSAB collaboratively identified target communities with the highest needs for interventions. The three target communities selected were MDC’s (i) Latino farm-working community, (ii) Latino low-income inner-city communities, and (iii) Latino LGBTQ+ community, as they were identified as being among the county’s most marginalized and underserved populations. The communities were identified through a collaborative process with the CSAB, whose members include community leaders, service providers, and researchers with extensive experience serving Latino populations in Miami-Dade County. Selection was based on community-identified needs, prior engagement activities, and collective assessment of populations perceived to be disproportionately affected by substance use, violence, HIV risk, and mental health challenges.

## 4. Methods

### 4.1. Recruitment and Data Collection

The community assessment involved a five-year process, with recruitment, data collection, and analysis conducted from 2018 to 2022. Participants were recruited via community recruiters using flyers, presenting at community meetings, leveraging community networks, and word of mouth in each community. A total of 97 community members, leaders, and key informants participated in the assessment plan, which was collaboratively established by the Community Engagement and Dissemination Core (CEDc) and the Community/Scientific Advisory Board (CSAB).

Eligibility criteria varied slightly by data collection activity. For example, community forum participants were required to be 18 years or older, self-identify as Latino/a (Latinx/e), and be members of the community being assessed. For focus groups, the same criteria applied, with additional inclusion of community leaders and individuals with relevant community involvement. For one-on-one interviews, participants were required to meet the same criteria and additionally hold leadership or management roles within community-based organizations serving the target populations. Across all activities, participants were required to be affiliated with the specific community under assessment.

Data collection was also guided by the principles of Community-Based Participatory Research (CBPR) and Grounded Theory (GT). Key CBPR principles informing this process included leveraging community strengths and resources, fostering collaboration among research partners, emphasizing community-defined problems, maintaining a long-term commitment to partnerships, and ensuring the dissemination of findings [54]. All data were collected using semi-structured guides and interview protocols developed in close collaboration with the Community/Scientific Advisory Board (CSAB).

Furthermore, the study employed methodological triangulation by collecting data across four distinct stakeholder groups: Community Forums, One-on-One Interviews, Focus Groups, and the CSAB. This multi-level approach allowed for the cross-verification of emerging themes, enhancing the trustworthiness of the findings and mitigating potential researcher bias [55].

### 4.2. Community Forums

Community forums have demonstrated effectiveness as a grassroots methodology for engaging rural community members in substance use prevention and intervention efforts. Palombi et al. [56] found that community forums, when planned in collaboration with researchers and community members, effectively increased overall awareness and knowledge of substance use within their respective target communities. Furthermore, forums rated highest by attendees were those featuring speakers from diverse professional backgrounds and cultural strengths [56]. The current study meticulously followed best practice data collection protocols using structured observation and the maintenance of standardized field notes during community forums [57].

To identify community needs, three community forums were conducted in 2018, with a total of 60 participants (*n* = 60) across the target communities identified by the CSAB. Each forum was co-facilitated by a faculty member and a CSAB member, and every step of the data collection process, including the development of semi-structured questionnaires, was collaboratively developed with community members of the CSAB. Venues were selected in collaboration with partnering community-based organizations (CBOs) from each respective community. In addition, CBOs were instrumental in recruiting participants who were active in their organizations. Forums were held in person, audio-recorded for accuracy, and guided by questionnaires that included questions such as: “*What kind of violence seems most prevalent in the community and who are the people most affected?*” and “*Can you think of resources in the community that are available for prevention and/or protection from violence?*” In addition to recordings, detailed notes were taken to annotate relevant themes.

### 4.3. One-on-One Interviews

To identify community resources, agency leaders were interviewed using semi-structured interview guides collaboratively designed with CSAB community members. These guides focused on the services provided by their respective agencies to the community. While semi-structured interviews are a valuable method for collecting rich, open-ended data, challenges can arise in engaging participants [58]. To mitigate challenges, interviews were conducted by experienced qualitative assessors skilled in building trust and rapport, engaging participants effectively, asking appropriate follow-up questions, and maintaining study focus without leading the respondents.

Following suggestions from CSAB community members, three agencies from each target community were selected and contacted for an interview, resulting in a total of nine interviewed agency leaders. The interview guides included illustrative questions such as: “*What services are most frequently utilized?*” and “*Which populations do you primarily serve?*” In addition to recorded responses, interviewers made relevant annotations during the interviews to capture non-verbal cues, contextual information, and emerging themes, which were subsequently included as part of the collected data.

### 4.4. Focus Groups

Several studies have cited the importance and similarities of using community forums and focus groups as data collection methods for community assessments; both enable in-depth discussions and insights from participants [59,60]. However, Cowley and Radford-Davenport [61] noted important distinctions justifying the use of both methods. While forum participants were often more likely to respond directly to each question, focus group participants tended to provide more in-depth and “off-topic responses,” particularly on topics specified by the researcher [62].

To explore deeper, in-depth perceptions of community needs, this study expanded upon the community forums with three focus groups (*n* = 20 total participants), with one group conducted for each target community. Participants were recruited using community outreach workers/recruiters who have established networks and relationships within each community. Recruiters used flyers, attended community meetings, and used word of mouth among their networks. Focus group data collection commenced and was completed in 2020. The first group, representing the Latino low-income inner-city community, was conducted in-person on the university campus. The second group, representing the Latino LGBTQ+ community, was conducted at one of the county’s most well-known LGBTQ+ community-based organizations and concluded on the last day before the United States declared COVID-19 a national emergency [63]. Consequently, recruitment and discussions for the third focus group, targeting the Latino farm-working community, were adapted to be conducted remotely in compliance with new COVID-19 physical-distancing mitigation strategies. Questions for the focus groups were also developed in collaboration with CSAB members and were informed by notes taken during the community forums and one-on-one interviews. Illustrative questions included: “*What are the most problematic substances in this community?*” and “*Who are the ones using these drugs?* (PROBE: youth, older adults, etc.)”. While formal pilot testing was not conducted, the guides were iteratively refined through CSAB input and early implementation to enhance clarity and relevance. The semi-structured guides are made available by the authors upon request.

### 4.5. Community/Scientific Advisory Board

The development of the Community/Scientific Advisory Board (CSAB) was a labor-intensive yet essential strategy for fostering sustained community partnerships. In fact, the establishment of community advisory boards has been recognized as a “mechanism for building capacity in the community and academic institution” [64]. To elicit feedback and evaluate their perceptions of the data collected from the five-year community assessment, CSAB members were asked to convene for an open group discussion, conducted over two separate evaluation meetings. During these meetings, all data collection strategies and preliminary results were presented and thoroughly discussed. Both meetings were audio-recorded for accuracy, and detailed notes were taken to annotate relevant themes.

### 4.6. Data Analysis

To ensure methodological rigor throughout the data analysis process, the study employed a systematic multi-level (four-level) sequential qualitative design, utilizing a Grounded Theory Approach [65]. The four distinct levels of data were from transcripts of all data collection sources including: (i) community forums conducted within the target communities; (ii) one-on-one interviews with agency leaders; (iii) focus groups, organized by target community; and (iv) a summary session with all community and scientific members of the CSAB (Figure 1). The analytic process followed a sequential and iterative approach, progressing from data familiarization to initial coding, codebook development, iterative refinement of codes, and ultimately the identification and integration of overarching themes.

Thematic analysis, recognized as one of the most widely used methods in qualitative research [66], was conducted manually. This involved repeated immersion in the data through careful reading and re-reading of interview transcripts and forum notes. All audio recordings were precisely transcribed, with each transcript independently reviewed for quality by a second transcriber. Following transcription, a digital repository was established to systematically organize the data by source, participant identification, and temporal sequence.

Given that many participants were Latinos, including recent immigrants, and often spoke in Spanish, transcripts were maintained in the participants’ original language throughout the analysis. All members of the analytic team were bilingual and bicultural, allowing coding and analysis to be conducted in participants’ original language. This approach preserved both semantic and cultural meaning and minimized potential translation bias. Quotations were translated into English solely for reporting purposes

Also guided by Grounded Theory (GT) principles, an initial bilingual coding framework was developed, allowing double-coding (where data excerpts could be assigned multiple relevant codes). This initial coding involved breaking down the data into smaller units to form codes that captured key concepts, categories, ideas, or themes emerging directly from the raw data. This process led to the development of a refined codebook and identifying overarching themes [67].

To enhance the validity and reliability of the analysis, inter-coder reliability checks were systematically employed throughout the coding process. Multiple members of the analytic team independently coded a subset of transcripts, and their coding decisions were then compared to identify discrepancies or differences in interpretation. Discrepancies were resolved through consensus discussions and peer debriefing sessions, to ensure consistent application of codes and strengthening the accuracy and credibility of the findings. For example, discrepancies arose when participant responses reflected multiple overlapping concepts (e.g., substance use and mental health coping). In such cases, coders discussed whether to refine code definitions or apply double-coding, ultimately allowing for multiple codes when appropriate to capture the multidimensional nature of the data.

While analysis was guided by Grounded Theory principles, it was not conducted in a purely inductive or tabula rasa manner. The analytic process was informed by sensitizing concepts related to substance use, violence, HIV risk, and mental health, as well as by the research team’s expertise and CBPR framework, allowing for an iterative interplay between emergent coding and conceptually informed interpretation.

Furthermore, theoretical saturation was rigorously pursued to confirm no new concepts or themes were emerging from the data. Saturation was also evaluated based on the consistency and depth of the identified themes across participant groups, ensuring that themes were sufficiently developed to provide a comprehensive and coherent understanding of the phenomena, with no major unexplained patterns remaining. Finally, the developed concepts and themes were coherently integrated to explain the identified needs and available resources within the target communities concerning substance use, violence, and HIV risk.

## 5. Results

**Figure 2:**
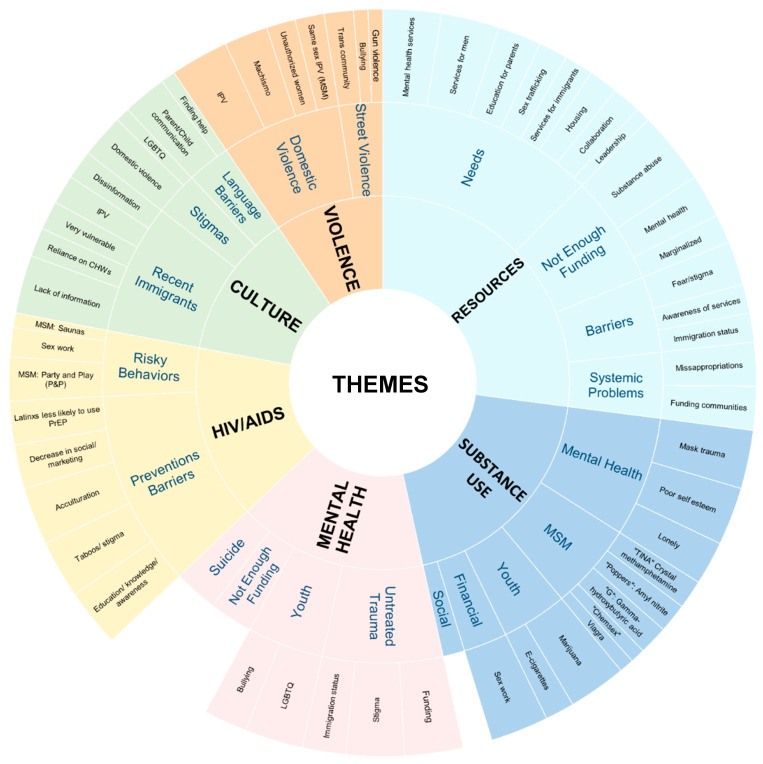
Thematic Coding.

This section presents the key findings from the five-year community assessment, highlighting identified needs and resources related to substance use, violence, HIV risk, mental health, and service availability across the three marginalized Latino communities in Miami-Dade County (See Figure 2. Thematic Coding).

Themes presented in Figure 2 were derived through an iterative coding process, as described in the Section 4.6, and reflect patterns that emerged across multiple data sources. While several themes were consistently observed across all three communities, others varied by community, reflecting distinct contextual experiences and needs. The prominence of themes in the figure was informed by the frequency with which themes appeared across the dataset; however, these counts are presented descriptively to illustrate the distribution and relative prominence of themes across participant groups and communities.

### 5.1. Substance Use

Following the onset of the COVID-19 pandemic in 2020, all assessed communities reported increased levels of substance use. Specifically, the farm-working community observed a rise in opioid, marijuana, and alcohol use.

Findings from the farm-working community forums indicated that youth were particularly vulnerable to substance use (mostly illicit drugs and alcohol). One farm-working focus group participants stated:

*“… the adolescents are using more marijuana and crack than the adults”*

Another participant in the same group noted:

*“Marijuana and crack with adolescents and adults too, but adults they involved in another kind of drugs… controlled medications… Percocet and all the psychotropics. Like uh, Xanax… Zoloft. I receive a lot of referrals with this problem, and I’m talking about children under 18.”*

Among inner-city groups, there was also a reported increase in vaping among youth. As one inner-city participant noted:

*“…vaping is rampant. vaping is the biggest problem that we’re seeing right now with the youth in the schools.”*

The LGBTQ+ community highlighted a significant increase in the use of methamphetamine, often referred to as “crystal meth,” “Tina,” or “T.” This increase was particularly noted during sexual gatherings known as “Party and Play” (PnP) or “chemsex,” which refers to the consumption of drugs to enhance sexual activities [67,68,69,70]. An LGBTQ+ focus group participant, who was also a CBO administrator, shared:

*“The culture of Tina is horrible… there’s houses in Miami, that is specifically tailor made to sell Tina, but also “orgy rooms”… because one of the things that Tina does is that it turn off your frontal lobe function, and increases your libido. So those people tend to be impulsive, they have sex for 72 h… gay guys are doing orgies and they go crazy and then they get raped and then crazy things happen and they end up here [at CBO] but what we’ve seen now is that the houses that sell the Tina, at the same time they rent rooms where the people that is already high, can enter and have sex. So now we’ve seen cases of sex trafficking, where the perpetrator make the person addicted to Tina, take the person to that houses, and then sell the person to other consumers in the same house and some of them are recorded and those videos are uploaded and put for sale. So it is just crazy.”*

These findings suggest distinct substance use patterns across the micro-communities, indicating the need for targeted interventions.

### 5.2. Violence

Discussions on violence revealed several salient issues within each community. Domestic violence emerged as the predominant concern in the farm-working community, followed by an increase in gun violence among youth. For example, two participants from the focus groups expressed: *“Domestic violence and sexual assault [occur] at the same time, most of the time… Most of the time is the couple… the partner.”*

Additionally:

*“There’s a gun problem here in Homestead. Everybody has a gun. We have a lot of cases, during this year I think, that I know of course, like seven kids died because of gun related, um, more than seven, gun related issues. But it was through violence, not through accidentally, through violence.”*

Within the LGBTQ+ community, sex trafficking was identified as a pervasive yet largely unaddressed issue. All three communities emphasized a lack of services addressing violence against men. A LGBTQ+ community leader expressed concern over the rise in violence and substance use within the community:

*“… we continued receiving victims of domestic violence, sexual assault in a very, huge numbers which, to be honest with you, it concerns me because I’ve been in this business for 12 years… not just cisgender women, but also transgender women. Sex trafficking and substance abuse; it’s really affecting this community, and I don’t see no one talking about it.”*

It is notable that these community-level reports of increased domestic violence contrast with data from the Florida Department of Health’s 2020 statistics [71], which indicate a steady decrease in domestic violence rates across Miami-Dade County since 2016. This disparity underscores the critical importance of conducting localized assessments to capture the nuanced needs of micro-communities that may not be evident in broader county-level data.

### 5.3. HIV Risk

Across all three target communities, there was a consensus on the urgent need for increased education to improve awareness of HIV risk behaviors and prevention strategies. Communities also noted a decline in HIV prevention awareness campaigns. The LGBTQ+ community made a direct connection between rising HIV infections and drug use, highlighting a crucial syndemic interaction within their population. Popular risky sexual activities, such as PnP/chemsex drug use among men who have sex with men (MSM), have been linked to sexual behaviors that increase the risk of HIV infection, other sexually transmitted infections (STIs) [72,73], and adverse mental health outcomes. The LGBTQ+ community noted that such parties are a prominent feature of the “Miami gay scene.”

While the farm-working community specifically emphasized the necessity of family-based interventions for HIV prevention. The participant statements below echo sentiments expressed across all three communities.

*“…the revenue that comes into this city comes from tourism and comes from that aspirational party lifestyle that Miami is known for, and specifically within the gay community. Miami is host to multiple circuit parties, multiple festivals that are like a hotbed for drug use and for substance abuse in general. And so the city of Miami pours a lot of money into hosting events like that and then to create, sort of continuing that culture, because they know that it brings a lot of money back into the city, but then they don’t invest back into the community so they don’t have like Community Centers for queer folks.”*

*“A lot of them are getting infected with HIV, so some of our new infections have to deal with “party and playing” [PnP] how they call it; a lot of them lose consciousness, just because of how severe the addiction might be.”*

*“… poor sexual health education in our schools is one of the factors that contributes to Miami first in the country HIV rate, right? But people don’t make the connections. You know, I think if parents knew the risks of their kids are exposed to, they’d be up in arms around having good sexual health education.”*

*“… a long time ago, many years… the community was more involved on providing prevention information like for HIV, like domestic violence. They were like, more active and now it’s like coming down… I don’t hear that many anymore like I used to do before. Like it’s shutting down now. There’s no more prevention meetings for all these like HIV, is like, I don’t know if they taking [it] like common daily living already?”*

### 5.4. Mental Health

Although mental health was not an initial primary focus of the study, it consistently emerged as a significant concern across all community assessment activities, warranting its inclusion in the findings. All three target communities expressed a clear need for increased mental health services and reported a rise in such needs since the COVID-19 pandemic.

A common theme reported by all groups was the tendency for mental health issues to be masked by substance use. One inner-city participants stated clearly:

*“…what I’ve seen is that people use substance abuse, to mask trauma. There’s a lot of trauma involved with living in poverty, like where’s your next meal coming from, issues with like stable housing, unsafe areas, so that’s what I’ve seen. So, a lot of children who live in poverty and have sort of all these very traumatic experiences, will use drugs to mask that. … and living in homes where there might not be any adult supervision. But I see mostly like trauma.”*

Furthermore, the farm-working community specifically reported increases in non-reported domestic violence, child sexual abuse, and suicide and suicide ideation. Yet, despite these pressing needs, the same community pointed out significant taboos surrounding the seeking of mental health services. One participant stated:

*“… on the mental health also, I think our culture is like, ‘I’m not crazy and I don’t have to go to a mental doctor.’ So is like more information to them what we need to provide”*

Key barriers to seeking services frequently cited by community members included stigma, judgement, and fears. One participant revealed:

*“… it requires more than one approach, and it requires an approach where we are understanding… the first thing that a client thinks cannot be that ‘I’m judged for what I’m saying’, that ‘I’m judged for what I’m doing’. They’re looking for help, and they need to know that if they’re coming to us for help, that’s what we’re here for. We’re not going to judge you, criticize you, or send you to ICE or immigration, or tell the police, or any of that… We’re here to help.”*

These findings are consistent with external data; the Substance Abuse & Mental Health Services Administration reported in 2020 [14] that Latinos are two times less likely to receive mental health services compared to non-Latino Whites; a finding based on access to services as well as cultural taboos.

### 5.5. Services and Resources

A pervasive theme across all three target communities was the urgent need for more services for substance abuse, violence/trauma, HIV prevention, and mental health services. A recurring observation was the limited availability of services specifically tailored for men.

*“They are reporting domestic violence against men… young men who were abused or they were involved in these domestic violence situation but they were the victims… I don’t think they [men] have the same kind of resources. I think there are more resources for women regarding violence.”*

Participants also identified systemic problems contributing to the unequal distribution of funds across communities, hindering effective service provision. One participant highlighted the relationship between data reporting and funding allocation.

*“You could be totally psychotic, and the funder will say, ‘Okay, that goes to a substance abuse treatment’, not a mental health bed. And the other thing that also happens is, whatever you report is your drug of choice is what our data tracks for the indigent population, right? So, if I say ‘No, I don’t drink that much. I just smoke marijuana’, but you’re an alcoholic. We don’t have that data to track. So, the funding and the data is always not good reports on what’s actually happening, particularly because it’s self-reporting.”*

*“…we get federal money and state money to work with victims, but the system, the legal system, they still don’t understand the connection between substance abuse and victimization.”*

Two participants made the case for the importance of assessing specific needs of micro-communities: *“I really do think it needs to be more narrowly focused on a community and not the entire ZIP code community.”* In the same group, another participant followed with: *“Walk four blocks, it’s another community, you got* [to] *engage completely differently. Same zip code, I mean same neighborhood, but they’re different.”*

Crucially, all communities concurred that collaboration among agencies is essential for comprehensively addressing the substance use, violence, HIV risk, and mental health (SAVA + MH) syndemic within and across the three communities.

*“it takes an entire, I would say a collaboration of organizations, you know, is really what it comes down to because we are all doing very similar work, but we’re doing it in silos. So, it’s a matter of bridging those gaps that all of us have to be able to really build those systematic changes that are needed in our community.”*

*“A policy change could be like with the funders to sort of force collaboration. So, when we did the substance abuse mental health service administration in Washington,* [and] *did their integrated program with HRSA, it worked really well. Because the money had to be that we have to work with the Department of Health.”*

## 6. Discussion

This community assessment, employing a community-based participatory research (CBPR) approach and Grounded Theory methodology over five years, provides crucial insights into the evolving substance use, violence, HIV risk, and mental health challenges facing marginalized Latino communities in Miami-Dade County. Our findings underscore the importance of localized, culturally informed assessments to identify syndemic relationships and tailor interventions, particularly in populations whose needs may be obscured by broader data.

The reported increases in diverse substance use patterns across the farm-working (opioids, marijuana, alcohol), inner-city (vaping), and LGBTQ+ (methamphetamine/PnP) communities post-COVID-19 pandemic highlight the profound impact of public health crises on vulnerability to substance use. The specific mention of “Party and Play” (PnP) or “chemsex” and its prevalence in the “Miami gay scene” among the LGBTQ+ community is particularly significant. This finding aligns with existing literature linking PnP drug use among men who have sex with men (MSM) to increased risk of HIV infection and other STIs [67,68,69,72,73]. This emphasizes a critical intersection of sexual health, substance use, and social environments within this micro-community, which necessitates integrated prevention and harm reduction strategies.

The nuanced findings regarding violence, particularly the prevalence of domestic violence in the farm-working community and sex trafficking in the LGBTQ+ community, underscore the heterogeneous nature of violence even within a shared ethnic group. The contrast between community-reported increases in domestic violence and declining county-level statistics for Miami-Dade County [71] may reflect differences in reporting patterns, particularly during the COVID-19 pandemic, as well as variations in data sources (e.g., arrest versus victimization data) and definitional differences across datasets. While this discrepancy is consistent with CBPR perspectives that emphasize the importance of community-defined problems, it should be interpreted with caution, as aggregated data may not fully capture localized experiences within specific communities. This underscores the potential value of localized, qualitative inquiry in identifying needs that may be less visible in broader surveillance systems. The consistent report across all three communities regarding the lack of services for violence against men further reveals a critical gap in current support systems. This gap may also be influenced by multiple factors, including cultural norms related to masculinity that may discourage reporting or help-seeking behaviors [74], as well as broader structural and reporting dynamics.

Our findings on HIV risk emphasize persistent educational gaps and a decline in awareness campaigns. The call for family-based interventions in the farm-working community speaks to the cultural context of prevention, aligning with approaches that leverage existing social structures, such as familismo, which emphasizes strong family ties and reciprocal support within Latino cultures [75]. The strong connection drawn by the LGBTQ+ community between HIV infections and drug use reinforces the syndemic concept, particularly in the context of PnP/chemsex, where drug use directly facilitates risky sexual behaviors [72,73] and adverse mental health outcomes. This reiterates the need for holistic interventions that simultaneously address substance use, sexual health, and mental well-being.

The prevalent and emergent theme of mental health needs, despite not being an initial study focus, highlights its relationship with substance use, violence, and HIV risk. The masking of mental health issues by drug use, coupled with the reported high suicide rates and cultural taboos against seeking mental health services in the farm-working community, often rooted in stigma and a reliance on informal support networks, underscores the unmet needs. These findings are consistent with existing literature indicating disparities in mental health service utilization among Latinos compared to non-Latino Whites [14]. The unreported domestic violence and child sexual abuse within the farm-working community further exacerbates mental health burdens, indicating a critical need for integrated trauma-informed care.

Collectively, results highlight a universal demand for increased and tailored services for substance use, violence/trauma, HIV prevention, and mental health. The identified barriers—such as insufficient services for men, prevalent stigma (often amplified by cultural values), fears of seeking help, and systemic issues in fund distribution—point towards structural inequities that impede effective community health. The unanimous call for collaboration among agencies resonates strongly with CBPR principles, suggesting that coordinated, multi-sectoral approaches are essential to effectively address the complex and interconnected substance use, violence, HIV risk, and mental health (SAVA + MH) syndemic identified across these vulnerable Latino communities.

While the current study does not aim for statistical representativeness, the inclusion of diverse Latino subpopulations and the use of multiple data sources enhance the transferability of findings to similar community contexts. Findings should be interpreted within the specific social and geographic context of Miami-Dade County.

### 6.1. Limitations

Despite its strengths, this study has several limitations that warrant consideration. First, as a qualitative study, its findings are context-specific and may not be directly generalizable to all Latino populations or other marginalized communities outside of Miami-Dade County. However, the depth of insight provided offers valuable transferable lessons. Second, while significant effort was made to ensure methodological rigor, including manual bilingual coding and multiple faculty reviewers, qualitative data analysis is inherently interpretative, and different researchers might emphasize different aspects of the data. Third, the shift to remote data collection for one focus group due to the COVID-19 pandemic, while necessary, may have influenced group dynamics or participation compared to in-person sessions. Finally, the reliance on self-report data means findings are subject to participant recall and potential social desirability bias, especially concerning sensitive topics like substance use, violence, and sexual behaviors.

### 6.2. Future Directions

Building on the findings of this study, particularly the identified service gaps, community-specific needs, and differences observed across the three target populations, the results offer several compelling avenues for future research and intervention. *(1) Develop and Evaluate Tailored Interventions:* Future research should focus on designing, implementing, and evaluating culturally and linguistically appropriate interventions for substance use prevention (e.g., vaping among youth, PnP harm reduction), domestic violence (especially for men), and HIV prevention (e.g., family-based programs that leverage cultural strengths like *familismo*). *(2) Explore Mental Health Service Uptake*: Given the significant unmet mental health needs, particularly the masking of issues by drug use and taboos in the farm-working community, future studies should explore innovative strategies to reduce stigma and increase access to mental health services within these specific cultural contexts. *(3) Leverage Technology for Community Assessment:* The labor-intensive nature of comprehensive qualitative community assessments, such as this five-year study, suggests a critical need for more efficient methodologies. Future research should explore the integration of advanced technologies like artificial intelligence (AI), sentiment analysis, and social listening tools to streamline and enhance the efficiency of data collection and analysis in community needs assessments. These technologies hold promise for identifying emergent themes, understanding community sentiment, and monitoring trends more rapidly, potentially allowing researchers to respond more dynamically to evolving community needs.

## 7. Conclusions

This five-year community assessment provides robust qualitative evidence of the intricate and interconnected health challenges faced by diverse marginalized Latino communities in Miami-Dade County. By privileging community voices through a rigorous CBPR approach, we have uncovered specific, localized indicators of substance use, violence, HIV risk, and mental health needs that often remain invisible in broader epidemiological data. The findings underscore the critical role of culturally competent and linguistically appropriate services, the urgent need to address the syndemic relationship between substance use, sexual health, and mental well-being, and the necessity of confronting systemic barriers to care. Ultimately, effective solutions require sustained, collaborative partnerships that leverage community strengths and address problems as defined by those most affected.

## Data Availability

The data presented in this study are available on request from the corresponding author.
